# Quzhou Aurantii Fructus Extract Attenuates Idiopathic Pulmonary Fibrosis by Regulating Nrf2/HO-1 Axis

**DOI:** 10.3390/biology15090716

**Published:** 2026-04-30

**Authors:** Li Yu, Lixia Wang, Jinyao Zhang, Ruimin Sun, Siming Zhang, Ping Yin, Ying Chen, Guocan Chen, Yiping Ye, Haitong Wan, Yu He, Yunxiang Chen, Lijiang Zhang

**Affiliations:** 1 Zhejiang Key Laboratory of High-Level Biosafety and Biomedical Transformation, School of Public Health, Hangzhou Medical College, Hangzhou 311305, China; yuli9119@126.com; 2 Key Laboratory of Drug Safety Evaluation and Research of Zhejiang Province, Center of Safety Evaluation and Research, Hangzhou Medical College, No. 587 Binkang Road, Binjiang District, Hangzhou 310053, China; 3 School of Basic Medical Sciences, Zhejiang Chinese Medical University, Hangzhou 310053, China; 4 Changshan Huyou Industry Development Center, Quzhou 324000, China; 5 School of Pharmacy, Hangzhou Medical College, Hangzhou 310013, China; 6 School of Pharmacy, Zhejiang Chinese Medical University, Hangzhou 310053, China

**Keywords:** QAFE, pulmonary fibrosis, bleomycin, inflammation, oxidative stress, Nrf2/HO-1 pathway

## Abstract

Recently, Quzhou Aurantii Fructus Extract (QAFE) was reported to exert anti-inflammatory effects on different types of respiratory diseases; however, it is yet to be determined whether it is effective in patients with idiopathic pulmonary fibrosis (IPF). The purpose of this study is to explore the capacity of QAFE to fight fibrotic disease, in particular how it works in relation to the regulation of the nuclear factor erythroid 2-related factor 2 (Nrf2)/heme oxygenase 1 (HO-1) pathway. QAFE was made with Quzhou Fructus Aurantii (QAF), and the content of four flavonoids in the samples prepared was analyzed by high-performance liquid chromatography (HPLC). The therapeutic effect of QAFE was experimented by establishing IPF models in mice and in cells. Identification of the mechanism of QAFE in IPF through knockout or knockdown of the Nrf2 gene. The experiments suggest that QAFE has the potential to prevent IPF-induced inflammation, collagen deposition, oxidative stress, and apoptosis of cells. Silencing Nrf2 by knockdown or knockout is enough to prevent the capacity of QAFE to inhibit the process of inflammation, oxidative stress, and collagen deposition to cause more serious lung injury and HO-1 expression downregulation. QAFE is a potential new antifibrotic drug in IPF with an effect on the Nrf2/HO-1 pathway that reduces inflammation and oxidative stress.

## Simple Summary

Idiopathic pulmonary fibrosis (IPF) is a progressive fibrotic lung disease, and the available medications face challenges in reversing its course. Traditional Chinese medicine has been given wide publicity because of its minimal side effects and enormous potential. Quzhou Aurantii Fructus Extract (QAFE) was used in this study, and through mouse and cell-based experiments, QAFE suppressed inflammation, scarring, and cell damage due to IPF by stimulating the Nrf2/HO-1 pathway. The study findings are that QAFE can be used as an antifibrotic agent in the treatment of IPF.

## 1. Introduction

Pulmonary fibrosis is a fibroproliferative respiratory disease associated with progressive and persistent fibrotic damage to the lung parenchyma, leading to symptoms that can include disruptive dry cough, increasingly severe dyspnea, shortness of breath on exertion, hypoxemia, bilateral diffuse infiltrative shadows, and eventual death as a consequence of the progressive deterioration of respiratory function [1,2,3]. Idiopathic pulmonary fibrosis (IPF) is a subtype of this disease that is largely characterized by a dry cough and dyspnea [4,5]. IPF incidence rates have continued to rise throughout the world each year owing to ongoing industrialization, the pollution of the environment, and population aging [5,6,7]. The drugs most commonly used to treat IPF include nintedanib and pirfenidone, but neither can effectively arrest or reverse disease progression in affected patients [8,9]. IPF can be effectively treated through lung transplantation, but limited donor availability, high treatment costs, and poor postoperative survival hamper the utility of this approach. Given these shortcomings, traditional Chinese medicine (TCM) has emerged as an attractive approach to relieving patient symptoms with few side effects [9,10], leading to growing interest in the research community.

Aurantii Fructus is the dried immature fruit of *Citrus aurantium* L. and its cultivated varieties. In July, the peel is harvested when it is still green, cut into two halves from the middle, and dried in the sun or dried at low temperature. Quzhou Aurantii Fructus (QAF) is a product that is produced by the drying of immature Citrus ‘changshan-huyou’ Y. B. Chang fruits, which are primarily found in Zhejiang, China. Nowadays, Changshan Huyou has developed more than 100 products in eight categories of “drink, food, health, beauty, medicine, fragrance, material, and tea”, and nearly 20 deep processing enterprises have emerged. The proportion of fruit deep processing has reached 45%, which has led to a 70% increase in the purchase price of fresh fruit. QAF was included in the “Zhejiang Provincial Chinese Medicine Concoctions Specifications (2015 Edition)” and the 2016 and 2018 “New Zhejiang Eight Essential Medicines” lists [11,12]. QAF is a medicinal and edible plant product. The primary bioactive components of QAF are flavonoids that include neohesperidin and naringenin, which reportedly exhibit beneficial pharmacological effects including antioxidant, antibacterial, immunomodulatory, hypolipidemic, and hypoglycemic activities. Li et al. found that QAF extract (QAFE) was able to engage the AMP-activated protein kinase (AMPK) and Mitogen-activated Protein Kinase (MAPK) signaling pathways to suppress inflammation in both acute lung injury models and LPS-treated RAW 264.7 macrophages [12]. Wei et al. further determined that the active ingredients in QAF were capable of protecting against lung fibrosis by inhibiting the stimulator of interferon genes (STING) pathway and mitigating inflammatory activity in a mouse IPF model system [13]. There are also several reports highlighting the hepatoprotective benefits of QAF in the context of mouse models of obesity and acute liver failure owing to its ability to inhibit inflammation and modulate the composition of the gut microbiota [14,15]. These studies have shown that QAFE has a good effect on pulmonary fibrosis and provides some experimental guidance for the further development of new methods for the treatment of pulmonary fibrosis.

Increasingly advanced studies focused on the pathological drivers of IPF have explored a range of factors including inflammation, autophagy, the polarization of macrophages, and oxidative stress [16,17,18]. Both inflammatory activity and oxidative stress are established as important negative factors that precede the onset of IPF [19,20]. The production of reactive oxygen species (ROS) in the lungs can provoke airway epithelial cell apoptosis and lead to the release of transforming growth factor beta (TGF-β) and various cytokines that promote myofibroblastic differentiation and the deposition of collagen while also further hampering antioxidant activity [21]. TCM preparations have shown promise for the prevention of pulmonary fibrogenic activity through the regulation of a range of pathways including the interleukin-6 (IL-6)-Janus kinase 2 (JAK2)-signal transducer and activator of transcription (STAT) 3/STAT1, MAPK, High Mobility Group Box 1 (HMGB1)/nuclear factor kappa B (NF-κB), and TGF-β1/Mothers against decapentaplegic homolog (Smad) 2/Smad3 pathways [22,23,24,25,26]. The 66 kDa nuclear factor erythroid 2-related factor 2 (Nrf2) protein is a key transcription factor involved in antioxidant enzyme regulation and consists of seven conserved Neh domains that are functionally important [27,28]. At baseline in the absence of oxidative stress, Nrf2 is retained in the cytosol through binding with Kelch-like ECH-associated protein 1 (Keap1), a negative regulator that interacts with the ETGE and DLG motifs present within the Neh2 domain, thereby rendering Nrf2 inactive. It then undergoes ubiquitination and subsequent 26S proteasome-mediated degradation, maintaining basal levels of antioxidant enzyme expression. In response to ROS or certain other stimuli, the oxidative modification of the cysteine residues of Keap1 leads to its consequent dissociation from Nrf2, thereby allowing this transcription factor to translocate into the nuclear compartment and promoting the upregulation of factors including heme oxygenase 1 (HO-1), glutathione peroxidase (GPx), catalase (CAT), and superoxide dismutase (SOD), bolstering antioxidant defenses in these cells [29,30,31]. Efforts to target this Nrf2/HO-1 signaling axis thus represent a major approach to combating oxidative stress. Whether QAFE can exert protective benefits against IPF through interactions with this Nrf2/HO-1 pathway, however, remains uncertain.

This study was developed to evaluate how QAFE treatment affects pulmonary fibrosis models in vitro and in vivo, with a focus on the underlying mechanisms of action. At a functional level, QAFE was found to protect against inflammatory activity and oxidative stress induced by intratracheal bleomycin instillation or the activation of fibroblasts with TGF-β1 through its ability to target the Nrf2 pathway. These results advance the current understanding of how QAFE can alleviate inflammation and oxidative stress, providing a foundation for its use in managing lung fibrosis.

## 2. Materials and Methods

### 2.1. QAFE Preparation

First, 50 kg of Quzhiqiao decoction pieces were added to 10 times the volume of water, soaked for 2 h, and then extracted by micro-boiling heating reflux 2 times, 1 h and 1 h, respectively. The extract was combined and collected after vacuum drying at 60 °C. Finally, dry powder was obtained (T230301).

### 2.2. HPLC Analyses of QAFE Flavonoid Content

After weighing out 100 mg of QAFE, it was combined with an appropriate amount of 50% methanol and placed in a 25 mL volumetric flask, and QAFE extraction was performed for 30 min at 40 °C in an ultrasonic bath. It was diluted with 50% methanol at constant volume after cooling and centrifuged at high speed for 10 min (12,000 rpm) to obtain the supernatant as the test solution. High-performance liquid chromatography (HPLC) analyses of these samples were conducted with an Agilent liquid chromatograph (Agilent Technologies, Santa Clara, CA, USA), allowing for the assessment of the hesperidin, neohesperidin, naringin, and narirutin content in these samples. Chromatographic separation was achieved with an Agilent Extend C_18_ (5 μm, 4.6 × 250 mm, Agilent Technologies, Santa Clara, CA, USA) column using a flow phase of acetonitrile–0.1% formic acid (18:82, *v*/*v*) and a 330 nm UV detection wavelength. External reference standards for each compound (hesperidin, neohesperidin, naringin, and narirutin) were used for the qualification of QAFE samples.

### 2.3. Animal Models

**Figure 1:**
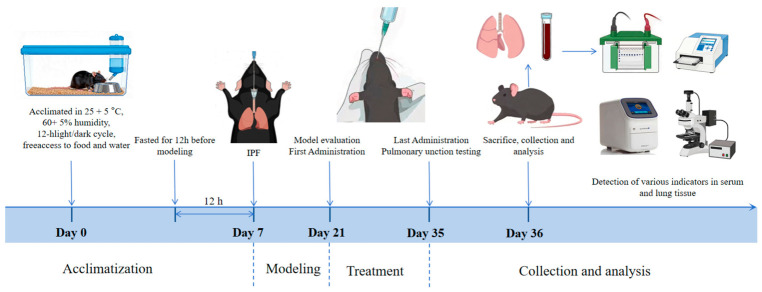
Experimental flow chart.

In total, 38 C57BL/6 mice (Production license number: SCXK (shanghai) 2022-0004) from Shanghai SLAC Laboratory Animal Co., Ltd. (Shanghai, China) were housed under controlled conditions (22 ± 2 °C, 50–60% humidity). To establish an IPF model, 31 mice were randomly selected and anesthetized with isoflurane, and then intratracheal injection of bleomycin (BLM, 2 U/kg) was performed in 200 μL of physiological saline [32,33]. Ten days later, one of the normal mice and one of the model mice were randomly selected for euthanasia, and the lung tissue was taken for H&E and Masson staining to verify whether the model was successful. The results (Figure S1) confirmed the presence of pulmonary fibrosis in the model group. Based on this validation, four days later (i.e., day 14 after intratracheal instillation of BLM), the remaining animals were randomly assigned to equally sized groups (*n* = 6 in each group), including control group, model group, low-dose QAFE (QAFE-L, 0.608 g/kg, i.g.), medium-dose QAFE group (QAFE-M, 1.216 g/kg, i.g.), high-dose QAFE group (QAFE-H, 2.432 g/kg, i.g.), and pirfenidone group (PFD, 200 mg/kg, i.g., MCE), with pirfenidone as the positive control. From that day on, selected doses of QAFE or PFD were delivered via oral gavage, repeating this process once per day for 14 days. Mice in the control and model groups instead received an equal volume of physiological saline. On day 14 post-administration, pulmonary function testing was conducted. The next day, mice were then euthanized with 5% isofluorane, and samples of blood, bronchoalveolar lavage fluid (BALF), and pulmonary tissue were collected. Similarly, six-week-old wild-type (WT) and Nrf2-knockout (Nrf2^−/−^) littermate male mice were purchased from Shanghai Model Organisms Center, Inc., (Shanghai, China) and used to assess the roles of QAFE-H in vivo. See Figure 1 for the specific experimental process. The Animal Care and Use Committee of Zhejiang Chinese Medical University approved all animal studies (approval No. IACUC-20231120-23).

### 2.4. Pulmonary Function Tests

Following the final round of QAFE or other treatment, a flexiVent small animal ventilator (SCIREQ, Montreal, QC, Canada) with non-invasive methods was used to evaluate various respiratory parameters for these mice, including functional residual capacity (FRC), peak expiratory flow rate (PEF), resistance of inspiration (RI), and dynamic pulmonary compliance (Cdyn).

### 2.5. BALF Analyses

To collect BALF samples, lungs were initially perfused with 1 mL of PBS, after which the lungs were lavaged three times and the fluid was collected. These BALF samples were centrifuged (10 min, 3000 rpm, 4 °C), and pellets were resuspended with 1 mL of PBS. Cells were counted with a hemocytometer (MC-6200Vet, MAXCOM, Shenzhen, China). Wright-Giemsa (Sigma-Aldrich, St. Louis, MO, USA) staining was used to detect inflammatory cells, counting 200 cells per slide.

### 2.6. ELISAs

Blood samples were harvested from the abdominal aorta, and serum was acquired following sample centrifugation. In addition, a 1 g sample of left lung tissue from each mouse was homogenized in 1 mL of PBS, and supernatants were harvested after centrifuging these samples (15 min, 12,000 rpm, 4 °C). Levels of BALF TNF-α (MM-0132M1, Meimian, Yancheng, China), IL-1β (MM-0040M1, Meimian, Yancheng, China), and IL-6 (MM-0163M1, Meimian, Yancheng, China), as well as lung tissue TNF-α, IL-1β, IL-6, total antioxidant capacity (T-AOC, MM-0743, Meimian, Yancheng, China), SOD (BC0170, Solarbio, Beijing, China), ROS (MM-43700, Meimian, Yancheng, China), MDA (BC0020, Solarbio, Beijing, China), MPO (MM-0338M1, Meimian, Yancheng, China), and HYP (MM-0308M2, Meimian, Yancheng, China) were analyzed with commercially obtained kits.

### 2.7. Histological and TUNEL Staining

After fixing lung tissue samples from the left lung for 48 h, these samples were cut into 4 µm sections. These sections were then subjected to hematoxylin and eosin (H&E) and Masson’s trichrome staining to assess tissue pathology and collagen deposition, respectively, conducting staining as directed by the manufacturer. TUNEL staining was performed as directed with an in situ cell death detection kit (11684795910, Roche, Basel, Switzerland) to evaluate cellular apoptosis, with nuclei being counterstained with DAPI. Tissues were then imaged via light microscopy (Nikon Eclipse Ci-L, Tokyo, Japan).

### 2.8. Immunohistochemistry (IHC)

IHC staining was used to measure Nrf2 and HO-1 levels within samples of pulmonary tissue. After harvesting lung tissues, fixing them, and cutting them into 4 μm sections, with these sections then being deparaffinized, rehydrated, blocked, and incubated overnight with antibodies specific for Nrf2 (1:100, AF0639, Affinity, Changzhou, China) or HO-1 (1:100, AF5393, Affinity, Changzhou, China) at 4 °C. Sections were then rinsed with PBS, incubated for 20 min with a secondary HRP-conjugated antibody (1:5000, ab97080, Abcam, Cambridge, UK), treated using 3,3′-diaminobenzidine (DAB), and mounted with neutral gum. A Nikon Eclipse Ci-L microscope (Nikon, Tokyo, Japan) was then used for imaging.

### 2.9. QAFE-Containing Serum Preparation

Male rats (6–8 weeks old) were randomized into control and QAFE treatment groups (*n* = 6/group). QAFE rats received intragastric QAFE treatment (2.432 g/kg, i.g.), while controls received an equivalent amount of physiological saline, with this treatment process being repeated once daily for 7 days. At 2 h after the final dose, rats were euthanized and blood was collected. Serum was then separated, inactivated, filtered through a 0.22 μm membrane, and stored at −80 °C or 4 °C for subsequent utilization.

### 2.10. Cell Culture, Transfection, and Grouping

The human fetal lung fibroblast-1 (HFL-1) cell line was obtained from iCell Bioscience Inc. (iCell-h017, iCell Bioscience Inc., Shanghai, China) and cultured in DMEM (41090-036, Gibco, Beijing, China) containing 10% FBS (FS301-02, TRANSGEN BIOTECH, Beijing, China) in a humidified 37 °C 5% CO_2_ incubator. GenePharma (Shanghai, China) synthesized siNrf2-#1 (5′-CGGCTTTGCGAAGTCATCCATCTCT-3′), siNrf2-#2 (5′-CGGTTGGCCCTTTCCTGCTTTATAG-3′), siNrf2-#3 (5′-CAAGGGACAGGTTGGAGCTGTTGAT-3′), and a negative control (si-NC, 5′-CACGUCUAGUAGUCGCUGATT-3′). Lipofectamine^®^3000 (L3000-008, Invitrogen; Thermo Fisher Scientific, Inc., Beijing, China) was used to transfect cells with these constructs. Earlier pharmacokinetic trials have also verified that following oral intake of 2.5 g/kg of Quzhike decoction in normal rats, the drug level in serum was determined. The findings showed that the flavonoid compounds (naringin, luteolin, hesperidin) were detectable in the plasma of the normal rats using the high-performance liquid chromatography analysis. The highest plasma concentrations of naringin, luteolin and hesperidin were 323.468, 566.970 and 428.512 5 g/L, respectively, and at the last time of detection at 6 h, the compounds were still at about half their maximum concentrations [34]. Thus, further in vitro experiments may use drug-containing serum as an experimental material. For experimentation, HFL-1 cells were assigned to the following groups: (1) a control group in which media was left unaltered for 48 h, (2) a TGF-β1 group treated for 48 h with 5 ng/mL TGF-β1 (HY-100558, MedChemExpress, Monmouth Junction, NJ, USA), (3) a QAFE-containing serum+TGF-β1 group (5 ng/mL TGF-β1, 20% QAFE-containing serum for 48 h), (4) a QAFE-containing serum+TGF-β1+si-NC group (si-NC transfection followed by 5 ng/mL TGF-β1 and 20% QAFE-containing serum treatment for 48 h), and (5) a QAFE drug-containing serum+TGF-β1+siNrf2 group (siNrf2 transfection followed by incubation with 5 ng/mL TGF-β1 and 20% QAFE-containing serum for 48 h).

### 2.11. MTT Assay

HFL-1 cell viability was assessed with an MTT assay kit (ST316, Beyotime, Shanghai, China). Briefly, HFL-1 cells were added to 96-well plates (4000/well), and then 10 μL of MTT reagent was added to each well. After a further 3 h of cell incubation, media was replaced with 100 μL of DMSO, and a CMaxPlus microplate reader (Molecular Devices, LLC, San Jose, CA, USA) was used to assess absorbance at 490 nm.

### 2.12. HFL-1 Cell Permeability Analyses

To assess the permeability of transfected HFL-1 cells, the cells were cultured on a transwell plate. Upon the TGF-β1 and QAFE drug-containing serum exposure, 100 μL of 1 mg/mL Fluorescein isothiocyanate-labeled 4-kDa dextran (FITC-D4, HY-128868, Sigma-Aldrich, St. Louis, MO, USA) in Hank’s solution was added in the insert and incubated in the CO_2_ incubator for 1 h at 37 °C. After that, 100 μL solution from the lower compartment of the plate was utilized to determine the fluorescence intensity under 485 nm excitation wavelength and 538 nm emission wavelength with a microplate reader. Permeability was calculated as relative fluorescent units (% baseline).

### 2.13. Flow Cytometry

Apoptotic cell death was analyzed by treating transfected HFL-1 cells for 48 h with TGF-β1 (5 ng/mL) and 20% QAFE-containing serum. These cells were then rinsed, harvested, and suspended for 15 min in 1 × binding buffer containing 5 μL Annexin V-FITC (556547, BD Biosciences, Franklin Lakes, NJ, USA) and 10 μL propidium iodide (556547, BD Biosciences, USA) at ambient temperature while protected from light. A FACSCalibur flow cytometer (BD Biosciences) was then employed to quantify apoptotic death, with the data being analyzed with FlowJo (v11.1.1, Tree Star, Ashland, OR, USA). Mitochondrial membrane potential (MMP) levels were assessed by staining cells with JC-1 solution (C2006, Beyotime, Shanghai, China). Following the resuspension of cells in PBS, they were analyzed with a FACS Calibur (BD Biosciences, Franklin Lakes, NJ, USA). Intracellular ROS levels were quantified via the treatment of these cells in the dark with 10 μM of DCFH-DA, and a flow cytometer (BD Biosciences, Franklin Lakes, NJ, USA) was then used to analyze the cells.

### 2.14. Intracellular MDA, SOD, and T-AOC Analyses

Following the treatment of HFL-1 cells, culture supernatants were harvested for analyses of IL-6, TNF-α, and IL-1β. Moreover, the cells were harvested, rinsed, and lysed in RIPA buffer (P0013B, Beyotime, Shanghai, China), with the supernatant being obtained following centrifugation. MDA (BC0020, Solarbio, Beijing, China), SOD (BC0170, Solarbio, Beijing, China), and T-AOC (MM-60148, Meimian, Yancheng, China) levels in these treated HFL-1 cells were determined with commercial kits according to provided directions.

### 2.15. Immunofluorescence

Following HFL-1 cell treatment as experimentally appropriate, these cells were rinsed, fixed, permeabilized, and treated for 2 h with 3% BSA for blocking. They were then probed overnight with rabbit polyclonal anti-Nrf2 (1:500, ab137550, Abcam, Cambridge, UK) and rabbit monoclonal anti-HO-1 (1:250, ab189491, Abcam, Cambridge, UK) at 4 °C. Secondary Alexa Fluor^®^ 555-goat anti-Rabbit IgG (1:1000, ab150078, Abcam, Cambridge, UK) was then used to treat these samples at ambient temperature for 1 h while protected from light, followed by counterstaining for 10 min with DAPI (Bioss). Anti-fluorescence quenching medium was employed to seal slides, which were imaged via immunofluorescence microscopy (Motic, Xiamen, China).

### 2.16. qPCR

Following HFL-1 cell transfection, an EZ-10 Total RNA Mini-Prep Kit (B618583, Sangon, Shanghai, China) was used to extract mRNA based on the provided directions, and 1 μg of RNA per sample was processed with a HiFiScript cDNA Synthesis Kit (CW2569, CWbio, Taizhou, China) to prepare cDNA. Then, a Hieff^®^ qPCR SYBR Green Master Mix (11201ES08, Yeasen Biotechnology (Shanghai) Co., Ltd., Shanghai, China) and a LightCycler 96 Real-Time PCR system (Roche, Switzerland) were used for qPCR analyses with primers from Sangon with the following sequences: Nrf2, Forward: 5′-ATTGCTGTCCATCTCTGTCAG-3′ and Reverse: 5′-GCTATTTTCCATTCCCGA GTTAC-3′; GAPDH, Forward: 5′-CCTCGTCCCGTAGACAAAATG-3′ and Reverse: 5′-TGAGGTCAATGAAGGGGTCGT-3′. Nrf2 expression was normalized to GAPDH via the 2^−ΔΔCT^ method.

### 2.17. Western Blotting

RIPA buffer (P1003B, Beyotime, China) was used to extract total proteins from cells and lung tissue samples, after which protein content was quantified through the BCA assay (pc0020, Beyotime). In total, 20 μg of protein per sample was separated via 10% SDS-PAGE, transferred to a PVDF membrane (Milipore, Billerica, MA, USA), and these blots were blocked for a 1 h period using 5% nonfat milk at ambient temperature, followed by primary antibody incubation overnight with antibodies specific for collagen I (1:1000, ab138492, Abcam, Cambridge, UK), collagen III (1:1000, ab184993, Abcam), α-SMA (1:1000, AF1032, affinity, Changzhou, China), fibronectin (1:1000, ab268020, Abcam, Cambridge, UK), Nrf2 (1:1000, ab62352, Abcam, Cambridge, UK), HO-1 (1:2000, ab189491, Abcam, Cambridge, UK), GAPDH (1:10,000, AMM22056N, Proteintech, Wuhan, China), and β-actin (1:2000, APR28873N, Proteintech, Wuhan, China) at 4 °C. The molecular weights of α-SMA and β-actin are both 42 kDa, so they overlap, but molecular weights of GAPDH (36 kDa) and HO-1 (33 kDa) are as well. Thus, GAPDH will be used as the inner reference to detect ECM markers, and 3-cloister will be used as the inner reference to detect Nrf2/HO-1. Then, secondary antibodies were incubated with blots for 1 h at ambient temperature, after which an ECL kit (Bio-Rad, Hercules, CA, USA) was used to image bands, with the Image J software (version 1.54p, National Institutes of Health, Bethesda, MD, USA) being leveraged for quantitative analysis.

### 2.18. Statistical Analysis and Software Used

The statistical software used to analyze the data was SPSS 20.0. When the measurement data of more than two groups were in the normal distribution and the test of homogeneity of variance met the conditions, the One-way-ANOVA single-factor analysis of variance was performed, and further comparison between the groups was by the Turkey test. In the event that it fits the normal distribution, but the variance is not evenly spread, Dunnett’s T3 test or independent-sample *t*-test is applied. When it fails to follow the normal distribution, the Kruskal–Wallis H test is employed. The significance level α = 0.05. All data were expressed as mean + standard deviation (s), and *p* < 0.05 served as the significance threshold. The images in this article are created or processed using Adobe Photoshop software (version 20.0, Adobe Inc., San Jose, CA, USA).

## 3. Results

### 3.1. QAFE Alleviates the Impairment of Pulmonary Function in IPF Model Mice

**Figure 2:**
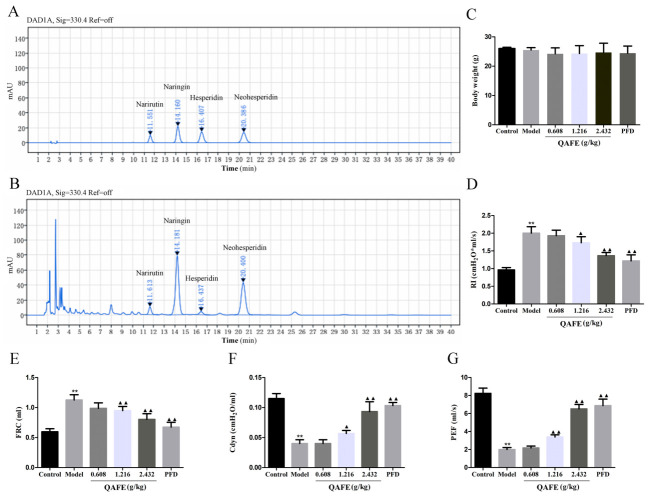
HPLC chromatograms for four chemical components in QAFE preparations and the impact of QAFE on the pulmonary function of IPF model mice. (**A**) HPLC chromatograms for reference compounds (hesperidin, neohesperidin, naringin, narirutin) and (**B**) QAFE at 330 nm. (**C**) Murine body weight in the indicated groups. (**D**–**G**) The resistance of inspiration (RI), pulmonary dynamic compliance (Cdyn), functional residual capacity (FRC), and peak expiratory flow rate (PEF) of IPF model mice with or without QAFE treatment. Data are means ± SD, *n* = 6. ** *p* < 0.01, vs. control; ^▲^ *p* < 0.05, ^▲▲^ *p* < 0.01, vs. model group.

The QAFE to be utilized in the study was a single batch of QAF extraction (batch number: T230301). This batch of extracts was used in all in vivo and in vitro experiments so that the batch variation would not impact the internal data comparability and reliability of the conclusion drawn of this study. Meanwhile, to guarantee the quality control of QAFE, this study has developed an initial quality control standard. HPLC was used to find the flavonoid content in QAFE samples. The concentrations of rutin naringin, naringin, hesperidin and neohesperidin 13.52, 128.48, 7.22 and 90.26 mg/g (Figure 2A,B) and the percentage of total flavonoids were 24.9%. The therapeutic benefits of QAFE treatment were next assessed in a BLM-induced mouse model of IPF. No significant differences in body weight were observed for the QAFE or PFD treatment groups (Figure 2C), and both of these treatments markedly decreased the RI and FRC of these mice together with the amelioration of Cdyn and PEF values relative to model mice (Figure 2D–G). Together, these data suggest that QAFE is capable of helping restore pulmonary ventilation in this BLM-induced model of fibrosis.

### 3.2. QAFE Protects Against Pulmonary Inflammation, Fibrosis, and Apoptotic Death in IPF Model Mice

**Figure 3:**
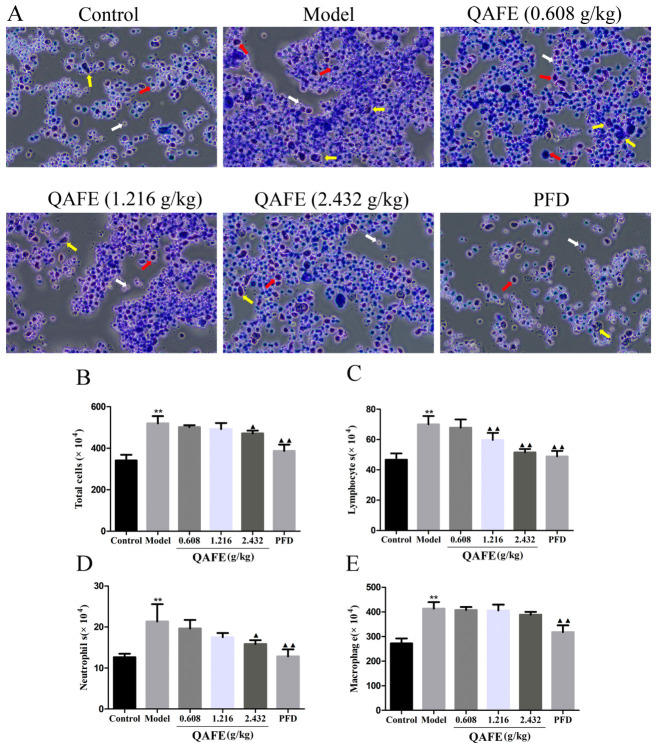
The impact of QAFE on airway inflammatory cell infiltration in IPF model mice. (**A**) Representative pictures of Wright-Giemsa staining (red arrows indicate the macrophages, white arrows indicate the lymphocytes, and yellow arrows indicate the neutrophils). Magnification: 20×. (**B**) Total inflammatory cells, (**C**) lymphocytes, (**D**) neutrophils, and (**E**) macrophages in BALF samples from the indicated groups. Data are means ± SD, *n* = 6. ** *p* < 0.01, vs. control; ^▲^ *p* < 0.05, ^▲▲^ *p* < 0.01, vs. model group.

**Figure 4:**
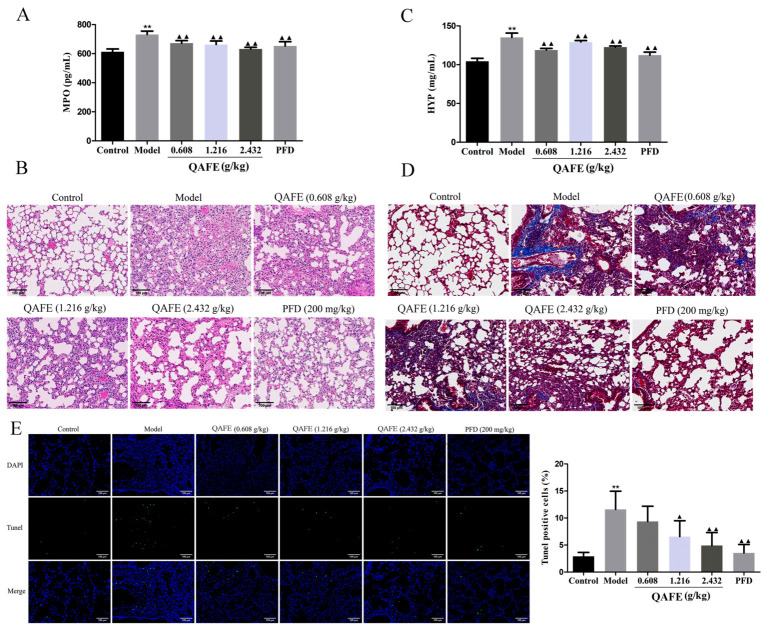
The impact of QAFE on pulmonary fibrosis and apoptotic death IPF model mice. Pulmonary tissue MPO (**A**) and HYP (**C**) levels were detected via ELISA. (**B**–**D**) H&E and Masson’s staining were used to assess the lungs of IPF model mice with or without QAFE treatment (magnification, ×200). (**E**) Representative TUNEL staining and corresponding quantification of lungs from IPF model mice with or without QAFE treatment (magnification, ×200). Data are means ± SD, *n* = 6. ** *p* < 0.01, vs. control; ^▲^ *p* < 0.05; ^▲▲^ *p* < 0.01, vs. model group.

BALF samples were harvested to probe the ability of QAFE to impact infiltration of the lungs by inflammatory cells (Figure 3A). Model group animals exhibited significant increases in BALF infiltration in terms of total cells, lymphocytes, macrophages, and neutrophils relative to controls (Figure 3B–E). Both QAFE and PFD treatment significantly reduced this inflammatory infiltration. In line with these findings, the levels of MPO, which is an established biomarker of neutrophil infiltration and activity, were markedly lower in the lung tissue samples from QAFE and PFD-treated mice (Figure 4A). H&E staining of lung histopathology indicated that control mice presented with normal lung tissue structures and the absence of alveolar septal thickening, while QAFE and PFD were sufficient to attenuate BLM-induced alveolar septum thickening, inflammatory cell infiltration, and the destruction of alveolar walls (Figure 4B). Collagen deposition was examined based on HYP levels in the lungs, revealing that QAFE treatment lowered HYP levels as compared to those for the model group (Figure 4C). Consistently, Masson’s staining demonstrated reductions in collagen fiber content in the QAFE and PFD treatment groups as compared to those in the lungs of BLM-induced IPF model mice (Figure 4D). TUNEL staining demonstrated that BLM induced extensive cellular apoptosis in the lungs, whereas QAFE and PFD reduced these levels of apoptotic death (Figure 4E). Together, these data underscore the ability of QAFE to protect against BLM-induced lung injury.

### 3.3. QAFE Suppresses Inflammation and Oxidative Stress in IPF Model Mice

**Figure 5:**
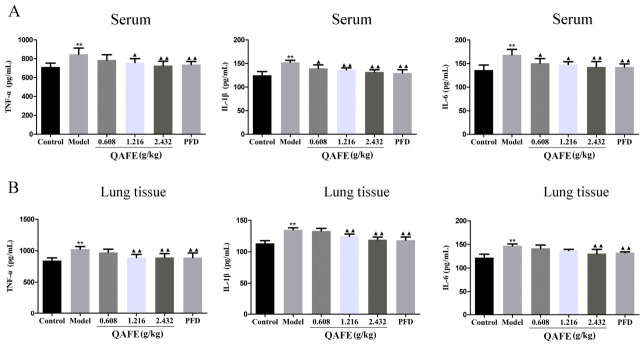
The impact of QAFE on the inflammatory cytokine levels in IPF model mice. ELISAs were used to measure TNF-α, IL-1β, and IL-6 levels in the serum (**A**) and lung tissues (**B**). Data are means ± SD, *n* = 6. ** *p* < 0.01, vs. control; ^▲^ *p* < 0.05, ^▲▲^ *p* < 0.01, vs. model group.

**Figure 6:**
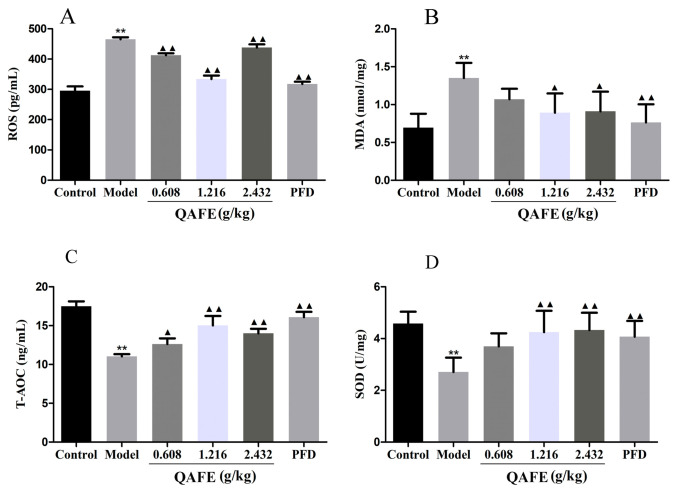
The impact of QAFE on oxidative stress in the lungs of IPF model mice. ROS (**A**), MDA (**B**), T-AOC (**C**), and SOD (**D**) levels were measured via ELISA in the lungs of IPF model mice. Data are means ± SD, *n* = 6. ** *p* < 0.01, vs. control; ^▲^ *p* < 0.05, ^▲▲^ *p* < 0.01, vs. model group.

ELISAs were employed to quantify inflammatory mediators and oxidative stress-related factors in serum and lung tissues from these experimental mice. BLM treatment was associated with significantly elevated TNF-α, IL-1β, IL-6, MDA, and ROS levels together with decreased SOD and T-AOC levels relative to control mice. QAFE and PFD treatments were both associated with reduced serum and lung TNF-α, IL-1β, and IL-6 levels (Figure 5A,B), together with decreased ROS and MDA levels and elevated T-AOC and SOD levels relative to the IPF model group (Figure 6A–D).

### 3.4. QAFE Activates Nrf2/HO-1 Signaling Activity in IPF Model Mice

**Figure 7:**
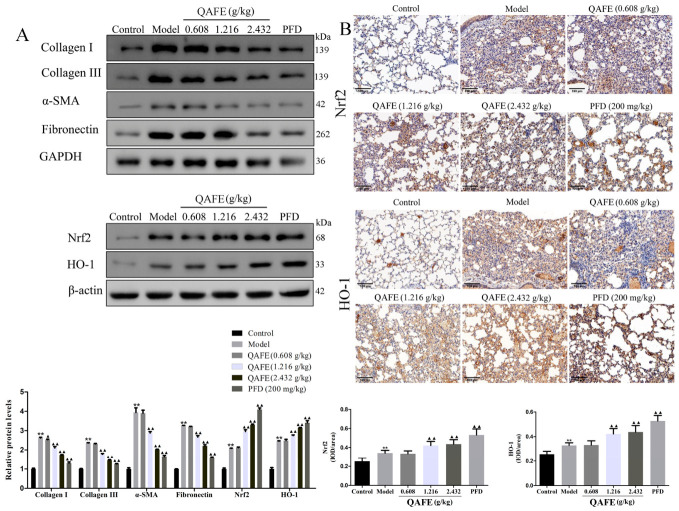
The impact of QAFE on fibrotic marker and Nrf2/HO-1 levels in IPF model mice. (**A**) Fibronectin, collagen I, collagen III, α-SMA, Nrf2, and HO-1 levels were assessed via Western blotting. (**B**) Nrf2 and HO-1 levels were detected via IHC. Scale bar, 100 μm. Data are means ± SD, *n* = 3. ** *p* < 0.01, vs. control; ^▲^ *p* < 0.05, ^▲▲^ *p* < 0.01, vs. model group.

To better understand how QAFE improves collagen deposition and oxidative stress, extracellular matrix (ECM) and Nrf2/HO-1 pathway-related protein levels were next analyzed. BLM treatment was associated with significant increases in fibronectin, collagen I, collagen III, α-SMA, Nrf2, and HO-1 protein content in the lungs of these mice (Figure 7A, original images are shown in Figure S2). Treatment with both QAFE and PFD reversed these changes, reducing levels of fibronectin, collagen I, collagen III, and α-SMA while upregulating Nrf2 and HO-1 relative to model group animals. IHC staining confirmed the ability of QAFE to induce higher levels of Nrf2 and HO-1 in the lungs of these BLM-treated IPF model mice (Figure 7B). These data confirmed that QAFE can protect against lung fibrosis in response to BLM instillation through Nrf2/HO-1 pathway activation.

### 3.5. Nrf2 Silencing Attenuates the Protective Benefits of QAFE-Containing Serum Treatment in TGF-β1-Treated HFL1 Cells

**Figure 8:**
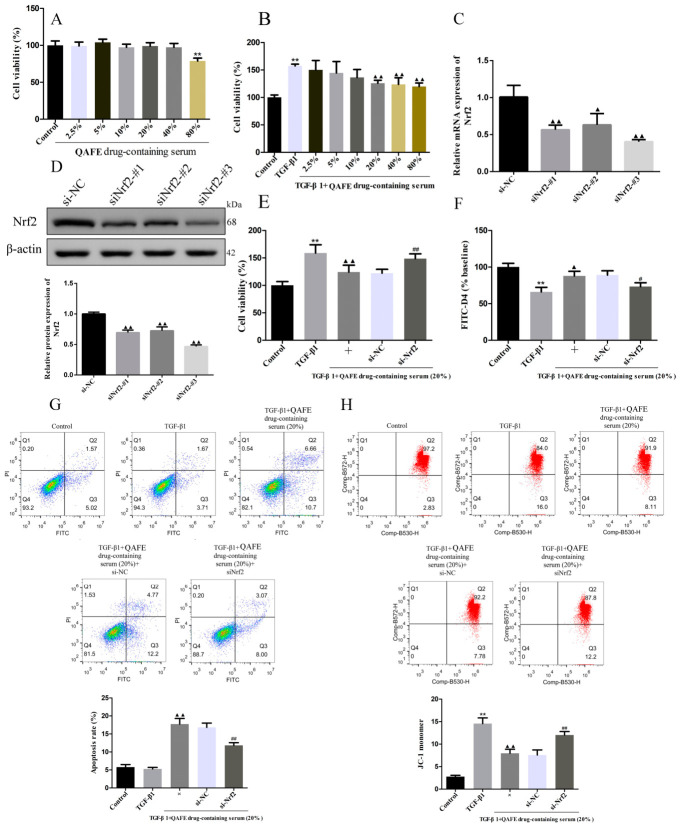
The impact of QAFE-containing serum on apoptosis and MMP levels in TGF-β1-treated HFL1 cells with or without Nrf2 silencing. (**A**,**B**) HFL1 cell viability without (**A**) or with (**B**) TGF-β1 treatment and with different QAFE-containing serum treatment concentrations was assessed through a CCK-8 assay, *n* = 6. (**C**,**D**) Nrf2 expression within HFL1 cells following Nrf2 silencing was assessed via qPCR and Western blotting. (**E**–**H**), Viability, permeability, apoptosis and MMP levels were assessed for TGF-β1-induced HFL1 cells in which Nrf2 had been silenced prior to treatment with QAFE-containing serum through CCK-8 (*n* = 6), FITC-D4 (*n* = 6), and flow cytometry approaches (*n* = 3). Data are means ± SD. ** *p* < 0.01, vs. control; ^▲^ *p* < 0.05, ^▲▲^ *p* < 0.01, vs. TGF-β1 or si-NC groups. ^#^ *p* < 0.05, ^##^ *p* < 0.01, vs. TGF-β1 + QAFE drug-containing serum + si-NC group.

**Figure 9:**
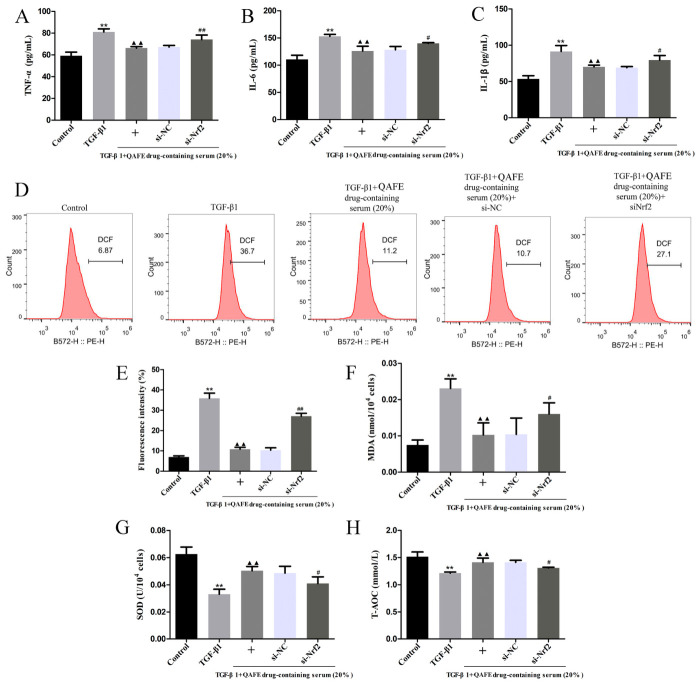
The impact of QAFE-containing serum on inflammation and oxidative stress levels in TGF-β1-treated HFL1 cells with or without Nrf2 silencing. (**A**–**C**) ELISAs were used to detect TNF-α, IL-1β, and IL-6 in supernatants from TGF-β1-induced HFL1 cells in which Nrf2 had been silenced, *n* = 6. (**D**,**E**) Flow cytometry was used to detect ROS levels in TGF-β1-induced HFL1 cells in which Nrf2 had been knocked down, *n* = 3. (**F**–**H**) ELISAs were used to assess MDA, SOD, and T-AOC levels in TGF-β1-induced HFL1 cells in which Nrf2 had been knocked down, *n* = 6. Data are means ± SD. ** *p* < 0.01, vs. control; ^▲▲^ *p* < 0.01, vs. TGF-β1 or si-NC groups. ^#^ *p* < 0.05, ^##^ *p* < 0.01, vs. TGF-β1+QAFE-containing serum+si-NC groups.

**Figure 10:**
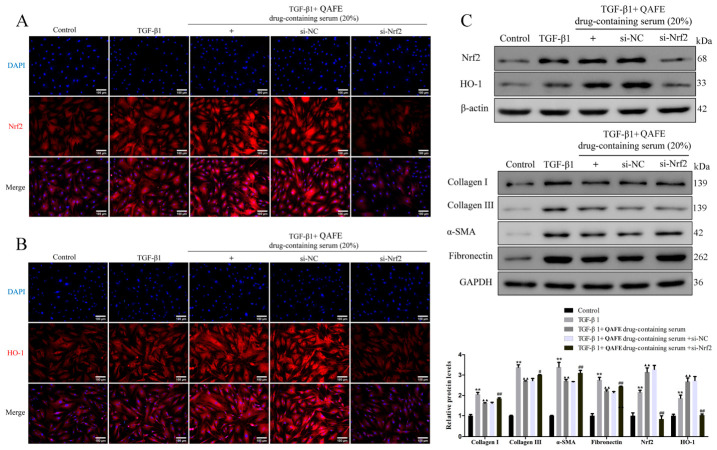
The impact of QAFE-containing serum on fibrotic and Nrf2/HO-1 pathway markers in TGF-β1-treated HFL1 cells with or without Nrf2 silencing. (**A**,**B**) Immunofluorescence was used to measure Nrf2 and HO-1 levels in TGF-β1-induced HFL1 cells in which Nrf2 had been knocked down with or without QAFE-containing serum treatment. Scale bar, 100 μm. (**C**) Nrf2, HO-1, collagen I, collagen III, α-SMA, and fibronectin levels were detected via Western blotting. Data are means ± SD, *n* = 3. ** *p* < 0.01, vs. control; ^▲▲^ *p* < 0.01, vs. TGF-β1 or si-NC groups. ^#^ *p* < 0.05, ^##^ *p* < 0.01, vs. TGF-β1+QAFE-containing serum+si-NC groups.

Through MTT analyses, the cytotoxicity of QAFE-containing serum was analyzed. Treatment with control serum or preparations of 2.5%, 5%, 10%, 20%, or 40% QAFE-containing serum did not impact HFL1 cell viability, although 80% QAFE-containing serum did significantly reduce cell viability (Figure 8A). However, treatment with 20%, 40%, and 80% QAFE-containing serum preparations resulted in significant reductions in the viability of TGF-β1-treated HFL1 cells (Figure 8B). Given these results, a 20% QAFE-containing serum concentration was selected for subsequent use. To probe the role of Nrf2 in TGF-β1-treated HFL1 cells, siRNAs specifically targeting this gene were developed, with siNrf2-#3 ultimately achieving the most significant Nrf2 downregulation (Figure 8C,D, original images are shown in Figure S3). MTT, FITC-D4, and flow cytometry approaches revealed that treatment with QAFE-containing serum significantly decreased the viability of these TGF-β1-induced HFL1 cells while promoting their permeability and apoptosis. Silencing Nrf2 reversed these effects of QAFE-containing serum on the viability, apoptosis, and MMP levels in these TGF-β1-treated HFL1 cells (Figure 8E–H). QAFE-containing serum exposure also significantly reduced the TNF-α, IL-1β, IL-6, ROS, and MDA levels in these TGF-β1-induced HFL1 cells while increasing SOD and T-AOC levels (Figure 9A–H). Western blotting and immunofluorescence staining revealed that these TGF-β1-induced HFL1 cells presented with increased levels of Nrf2 and HO-1 expression together with reductions in levels of fibronectin, collagen I, collagen III, and α-SMA (Figure 10A–C, original images are shown in Figure S4). Silencing Nrf2 reversed these beneficial effects of QAFE-containing serum treatment (Figure 9 and Figure 10). These data support a model wherein exposure to QAFE-containing serum can induce the apoptotic death of TGF-β1-treated HFL1 cells through a mechanism at least partially dependent on Nrf2 upregulation and activity.

### 3.6. Nrf2 Deficiency Exacerbates BLM-Induced IPF and Attenuates the Protective Influences of QAFE

**Figure 11:**
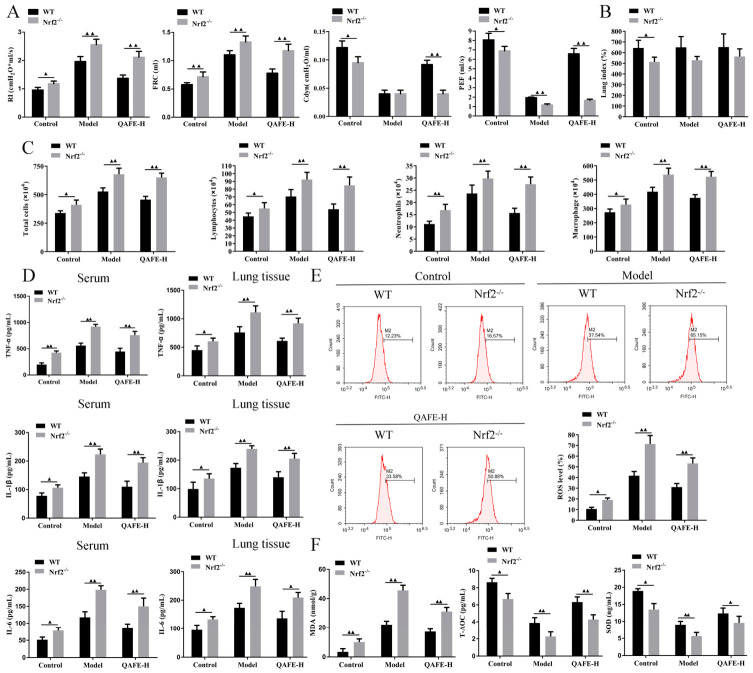
The impact of QAFE on pulmonary function, inflammation, and oxidative stress in BLM-induced WT and Nrf2^−/−^ mice. (**A**) Pulmonary function test of RI, FRC, Cdyn, and PEF, *n* = 6. (**B**) Lung index of BLM-induced WT and Nrf2^−/−^ mice with QAFE treatment, *n* = 6. (**C**) Total inflammatory cells, lymphocytes, neutrophils, and macrophages in BALF samples from QAFE-treated IPF mice with Nrf2 deficiency, *n* = 6. (**D**) Levels of Inflammatory factors (TNF-α, IL-1β, and IL-6) in serum and lung tissue samples were detected by the ELISA kits, *n* = 6. (**E**) Flow cytometry analyses of ROS levels in lung tissue samples, *n* = 3. (**F**) The levels of oxidative stress markers such as MDA, T-AOC, and SOD were measured with ELISA kits, *n* = 6. Data are means ± SD. ^▲^ *p* < 0.05, ^▲▲^ *p* < 0.01.

**Figure 12:**
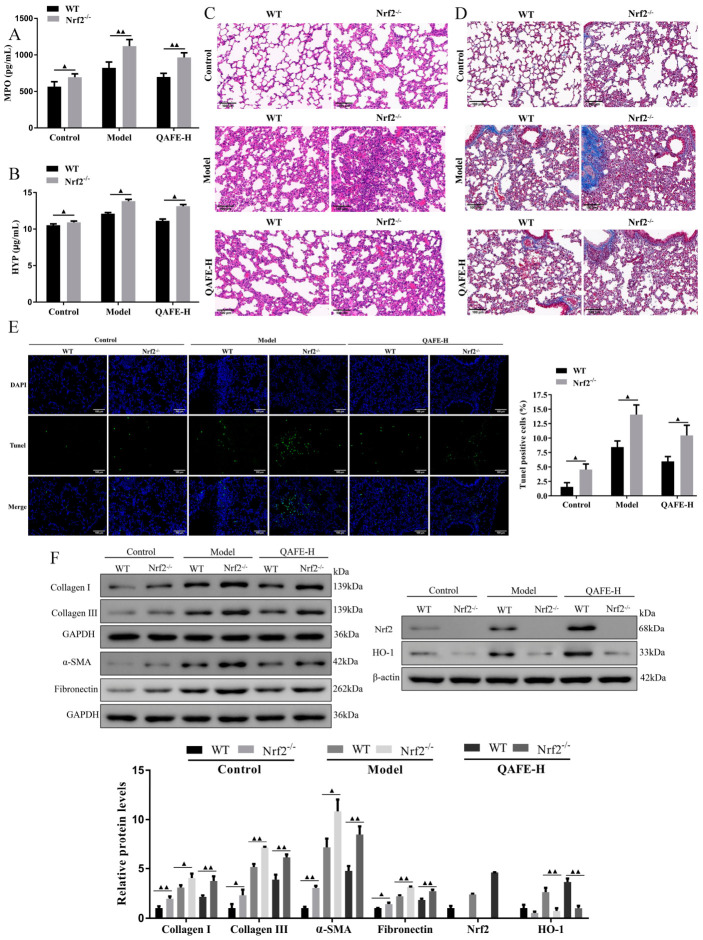
The impact of QAFE on collagen content and apoptosis within the lung tissue of BLM-induced WT and Nrf2^−/−^ mice. The levels of (**A**) MPO and (**B**) HYP expression after QAFE treatment in BLM-induced WT and Nrf2^−/−^ mice, *n* = 6. (**C**) Pathological damage of lung tissue measured by H&E staining (Scale bar, 100 μm), *n* = 6. (**D**) Collagen in the lung tissue samples was observed by Masson’s staining (Scale bar, 100 μm), *n* = 6. (**E**) Representative images of apoptotic cells in TUNEL staining from BLM-induced WT and Nrf2^−/−^ mice with QAFE treatment (Scale bar, 100 μm), *n* = 6. (**F**) Collagen I, collagen III, α-SMA, Fibronectin, Nrf2, HO-1, GAPDH, and β-actin protein expression within lung tissue of BLM-induced WT and Nrf2^−/−^ mice were detected by Western blotting, *n* = 3. Data are means ± SD. ^▲^ *p* < 0.05, ^▲▲^ *p* < 0.01.

To further verify the effect of QAFE in BLM-induced IPF by targeting Nrf2, WT and Nrf2^−/−^ mice were treated with BLM and QAFE. As expected, Nrf2 deficiency significantly exacerbated the pulmonary function, inflammation, and oxidative stress in the lung tissues of IPF mice (Figure 11). In addition, Nrf2 deficiency further increased BLM-induced collagen deposition and cell apoptosis, as evidenced by the increase in ELISA assays, Masson’s staining, and TUNEL-positive cells (Figure 12A–E). As shown in Figure 12F (Original images are shown in Figure S5), Nrf2 deficiency remarkably enhanced the BLM-induced upregulation of collagen I, collagen III, α-SMA, and Fibronectin in lung tissues, suggesting that Nrf2 defends lung tissues from BLM-induced pulmonary fibrosis. More importantly, the Nrf2 knockout attenuated the protective effects of QAFE-H (2.432 g/kg) in BLM-induced Nrf2^−/−^ mice (Figure 11 and Figure 12). Taken together, these findings showed that the regulatory functions of QAFE on BLM-induced pulmonary fibrosis are at least partially through the activation of the Nrf2/HO-1 pathway.

## 4. Discussion

QAF, also referred to as Changshanhuyou, has long been used as a traditional medicinal preparation in Changshan County, Quzhou City, Zhejiang Province in China. QAF has previously been demonstrated to be a rich source of flavonoids including neohesperidin, narirutin, hesperidin, and naringin, with these compounds exerting anti-diabetic and anti-obesity functions [15,35,36]. QAF is a good variety of tangerine pomelo selected from the natural hybrid progeny population of sour orange and pomelo. It is a national geographical indication product. Xiaoqingguo is the raw material for processing traditional Chinese medicine “QAF”. From small green fruit to “QAF”, from golden fruit on the tree to pomelo juice, pomelo has become the rich ‘Jinguo’ of the people in Changshan. It is reported that this year, the total output of Changshan Huyou small green fruit slices is expected to exceed 6000 tons, with a total output value of more than 100 million yuan.

Work from our group has also demonstrated that hyperlipidemia-associated oxidative stress can also profoundly impact the pharmacokinetic and pharmacodynamic properties of a QAF decoction [34]. The drug studied in this study is the crude extract of QAF, which has not been enriched with macroporous resin. And according to relevant data reports, the components contained in QAF are flavonoids [11,12]. Here, HPLC analyses were used to assess the flavonoid levels in QAFE samples, providing guidance for efforts to standardize QAFE quality control. A growing number of studies have attested to the beneficial effects of QAF treatment for various respiratory ailments [13]. The dose of QAFE in mice was determined by preliminary studies and experiments of our research group [11,12]. This and in combination with the earlier results of the H&E staining led to the selection of the doses of QAFE in mice; 0.608 g/kg (low dose), 1.216 g/kg (medium dose) and 2.432 g/kg (high dose) as the three doses of QAFE to be used in future studies. In the past, research indicated that the extraction rate of QAF was approximately 35.3% [11]. The clinical dose of human beings was 10 g QAF [37]. The equivalent dose of QAFE in mice was approximately 0.77 g/kg, and the high dose was approximately 3 times of the equivalent dose, which was in the reasonable range of exploratory pharmacodynamic study, according to the body surface area. Also, our former experiment on the maximum dose of QAFE in mice indicated that no side effects were noted despite the maximum dose of 100 g/kg [38]. Thus, the drug dose in the present study was a safe dose in the mice. This study was thus devised to explore the value of QAFE as a prophylactic agent capable of protecting against pulmonary fibrosis and preserving lung health through a detailed investigation of its antioxidant and anti-inflammatory effects in vitro and in vivo.

Intratracheal BLM instillation is the most common approach to establishing animal models of IPF in order to explore the treatment and etiology of this disease, providing a stable model system with clearly defined stages of fibrotic progression that are a good representation of human disease [38,39]. The literature has found that the peak of the fibrosis response in mouse tissues occurred on day 14 after intratracheal administration of BLM [40]. Thus, this experiment decided to initiate administration on day 14 to determine the therapeutic impact of the drug in the already formed fibrosis but not the pre-protective impact [41,42]. Meanwhile, despite fibrosis induced by BLM starting to spontaneously reverse by the 28th day, all experimental groups in this study were euthanized at the same time and the samples were harvested. Thus, all of the experimental groups were subject to the same impact of the spontaneous regression of the fibrosis induced by BLM. The pathological level was equal in all the model mice; hence, the spontaneous regression period did not affect the outcome of the experiment. Accordingly, BLM was herein used to establish a murine IPF model system, and these animals were treated with QAFE. To gain initial insight into the effects of QAFE on pulmonary fibrosis-related symptoms, changes in murine body weight, respiratory function, inflammatory activity, and key biochemical or physiological indices associated with oxidative stress were evaluated. Progressive dyspnea is a hallmark of pulmonary fibrosis that profoundly affects patient quality of life [9,43,44]. This dyspnea is primarily the result of the replacement of healthy pulmonary tissue with extracellular matrix components and fibrous foci, destroying the normal alveolar structures such that lung compliance, ventilation, and air exchange are all compromised [45,46]. Here, QAFE was found to offer significant protective value through its ability to reverse BLM-induced changes in lung functional parameters including compliance, lung capacity, and tidal volume. This suggests that QAFE intervention can effectively remediate the dyspnea resulting from pulmonary fibrosis.

In prior studies, BLM was shown to induce inflammatory and fibrotic activity in pulmonary tissues, with intratracheal BLM dosing resulting in the upregulation of profibrotic cytokines [47,48]. Here, following the administration of BLM, both H&E and Masson’s staining revealed significant thickening of the alveolar septa and extensive collagen deposition in mice. These changes coincided with clear evidence of inflammation in the resected pathological specimens, higher levels of inflammatory cell infiltration, and elevated inflammatory cytokine (IL-1β, IL-6, and TNF-ɑ) levels in pulmonary tissues and serum. QAFE treatment, in contrast, effectively reversed these adverse inflammatory changes in BLM-treated mice, reducing these cytokine levels and minimizing the evidence of inflammation in pathological sections prepared from the lungs of treated mice. HYP levels have been reported to reflect collagen content in the lungs such that they can be used to quantify the extent of pulmonary fibrosis [49,50]. Both ɑ-SMA and collagen III are key fibroblast activation markers indicative of the myofibroblastic transformation of fibroblasts [50]. Here, mice in the BLM treatment group presented with significant increases in HYP, ɑ-SMA, collagen I, collagen III, and fibronectin protein expression, while QAFE administration effectively lowered these levels, providing direct evidence that QAFE can alleviate pulmonary fibrosis.

The loss of appropriate redox homeostasis is a key component of the pathogenesis of IPF [51]. After the lungs sustain an initial injury, the accumulation of high levels of ROS can trigger the peroxidation of lipids at a level beyond the capacity of the local antioxidant system to buffer, disrupting redox balance and promoting the release of inflammatory cytokines and profibrotic growth factors that drive myofibroblast activation and the consequent induction of pulmonary fibrosis [52,53]. Efforts to reverse this imbalance between oxidative stress and antioxidant activity are thus vital for the inhibition of IPF. In the present study, oxidative stress was quantified through assays focused on ROS, MDA, T-AOC, and SOD levels and activity. Animals in the BLM group presented with elevated ROS and MDA levels together with significant reductions in SOD and T-AOC, whereas QAFE effectively reversed these undesirable changes. This suggests that QAFE can protect against oxidative stress driven by bleomycin exposure, ultimately limiting the progression of pulmonary fibrosis.

The findings indicated that on some of the test outcomes, the medium dose of the test was as effective as the high dose in improving the detection of serum inflammatory indicators. This could be because flavonoid glycosides have a relatively high relative molecular mass, water solubility and fat solubility are not good and the gastrointestinal absorption is carried by transporters mainly [54]. Achieving saturation of these transporters and metabolic enzymes with the medium dose may be the reason that there is not much difference between the medium dose and high dose of QAFE with respect to effective absorption of these transporters and metabolic enzymes, which results in a platform period of improvement effect of medium- and high-dose QAFE on certain serum inflammatory indicators, and there was no statistical difference between some model groups. In the detection of oxidative stress in lung tissue, the improvement effect of the medium dose on ROS and T-AOC was better than that of the high dose, which may be due to the redox cycle of high-dose flavonoids, the generation of quinones, and the catalytic Fenton reaction to produce ROS [55,56]. Nevertheless, its ROS is not sufficiently high to influence its therapeutic action. Thus, the effect of the high dose on certain oxidative stress indexes is not as strong as the effect of the medium dose; however, the effect on pulmonary fibrosis of the high dose is still good in terms of antioxidant activity. The high dose was found to be effective in all indicators based on all the experimental results of drug dose screening. Meanwhile, in BALF samples, low and medium doses did not significantly affect the enhancement of inflammatory cells. Thus, we selected the high dose as the dose in the future Nrf2 knockout experiments.

TGF-β is central to the pathogenesis of IPF, functioning in part by driving high levels of ROS production [57]. When myofibroblasts produce excessively high levels of ROS, this can lead to alveolar epithelial cell apoptosis, driving the progression of pulmonary fibrosis in a debilitating feedback cycle [52,53]. TUNEL staining experiment in vivo demonstrated that the rate of apoptosis of the lung tissue cells in the model mice treated with BLM was much higher and the ratio of apoptotic positive cells was much lower after QAFE treatment, suggesting that QAFE could be an effective antidote of apoptosis induced by bleomycin in alveolar epithelial cells. Flow cytometry analysis of the HFL1 cells activated by TGF-β1 in vitro further demonstrated that following the treatment of the cells with QAFE-containing serum, the percentage of positive cells rose, and the cell viability reduced, which showed that QAFE-containing serum could induce apoptosis of the activated fibroblasts. But in the case of normal HFL1 cells, not subjected to the TGF-β1 stimulation, QAFE-containing serum treatment did not suppress cell viability. This finding indicates that QAFE is able to induce apoptosis of fibrotic cells and save bleomycin in alveolar epithelial cells, thus diminishing the fibrotic process.

Nrf2 is a central transcription factor in the antioxidant system [58]. Research has indicated that significant production of ROS is observed in the lungs in a pathological condition, which worsens tissue destruction and develops fibrosis. High concentrations of ROS will alter the cysteine residues of Kelch-like ECH-related protein 1 (Keap1), alter conformation, make Nrf2 release into the nucleus, and bind to ARE to induce downstream HO-1 expression [59,60]. This is aligned with experimental findings that the Nrf2 and HO-1 protein levels are increased with the treatment of BLM alone, which is the endogenous antioxidant defense mechanism instigated by the body in the case of oxidative damage. However, experimental findings revealed that this compensatory upregulation was not sufficient to overcome oxidative stress caused by BLM. The amount of oxidative stress and fibrosis in the model group remained significantly greater than that in the normal group. Fibrosis progression could not be prevented by the use of endogenous compensatory antioxidant. Following QAFE intervention, Nrf2 and HO-1 expression in the QAFE group compared to the model group was further enhanced, and inflammation, oxidative stress and fibrosis caused by BLM were greatly suppressed. This finding indicates that QAFE has the potential to induce the Nrf2/HO-1 pathway further using the premise of endogenous compensation, such that its antioxidant potential is adequate to negate and revert oxidative stress caused by BLM, thus mitigating pulmonary fibrosis damage. To confirm the importance of Nrf2 in the protective antifibrotic activity of QAFE, this gene was silenced, abrogating the protective effects of this extract in the TGF-β-induced HFL1 cell model system. Moreover, Nrf2 knockout weakened the lung protective effects of QAFE in BLM-induced Nrf2^−/−^ mice. Accordingly, these results suggested that Nrf2 was capable of mediating the antifibrotic and antioxidant effects associated with QAFE treatment.

Nevertheless, there are also some limitations of this study. In spite of the fact that intratracheal instillation of BLM is the most popular animal model of IPF, it does not perfectly mimic human IPF. Thus, the results of the BLM model should be translated into clinical practice with caution. However, this model remains a valid model in assessing the drug treatment of pulmonary fibrosis [61,62,63,64]. To further confirm the effectiveness of QAFE, a model more resembling the pathological mechanism of human IPF should also be used to do experiments, like multiple low-dose BLM administration or Ad-TGF-β1 adenovirus induction [65,66]. Meanwhile, our research on the mechanism is certainly insufficient, in particular regarding the absence of direct evidence of Nrf2 nuclear translocation and experimental evidence of the interaction of Nrf2 with profibrotic pathways, including TGF-β/Smad. Nonetheless, it has been established that nuclear translocation of Nrf2 can augment antioxidant defenses and block ROS, thus preventing ROS activation of the TGF-β/Smad signaling pathway, thus blocking TGF-β/Smad-induced profibrotic actions [67,68]. Thus, the follow-up study will further investigate the exact mechanism of action of QAFE on Nrf2 and the TGF-β/Smad pathway in order to better implement QAFE in clinical practice.

## 5. Conclusions

In summary, these results highlight the ability of QAFE to exert anti-inflammatory and antioxidant activity in both murine and cell-based models of IPF through its ability to activate Nrf2 signaling, arresting fibrotic disease progression. These data underscore the therapeutic promise of QAFE as a tool for IPF treatment.

## Data Availability

Data will be made available on request.

## Supplementary Materials

The following supporting information can be downloaded at: https://www.mdpi.com/article/10.3390/biology15090716/s1, Figure S1: Comparison of H&E staining and MASSON staining between normal and IPF rat lung tissues (200×, 100 μm; 400×, 50 μm); Figure S2: The original stripe in Figure 7; Figure S3: The original stripe in Figure 8; Figure S4: The original stripe in Figure 11; Figure S5: The original stripe in Figure 12.

## Abbreviations

Abbreviations	Definition
BALF	Bronchoalveolar lavage fluid
BLM	Bleomycin
CAT	Catalase
Cdyn	Dynamic pulmonary compliance
DAB	3,3′-diaminobenzidine
ECM	Extracellular matrix
FRC	Functional residual capacity
HFL-1	Human fetal lung fibroblast-1
HO-1	Heme oxygenase 1
HPLC	High-performance liquid chromatography
IPF	Idiopathic pulmonary fibrosis
MMP	Mitochondrial membrane potential
Nrf2	Nuclear factor erythroid 2-related factor 2
PEF	Peak expiratory flow
PFD	Pirfenidone
QAFE	Quzhou Aurantii Fructus extract
RI	Resistance of inspiration
ROS	Reactive oxygen species
SOD	Superoxide Dismutase
TCM	Traditional Chinese medicine
TGF-β	Transforming growth factor beta
