# Supplementary figures and images for: Quzhou Aurantii Fructus Extract Attenuates Idiopathic Pulmonary Fibrosis by Regulating Nrf2/HO-1 Axis
Source: Biology (Basel). 2026 Apr 30;15(9):716. doi: 10.3390/biology15090716 (PMC13162770; doi:10.3390/biology15090716)

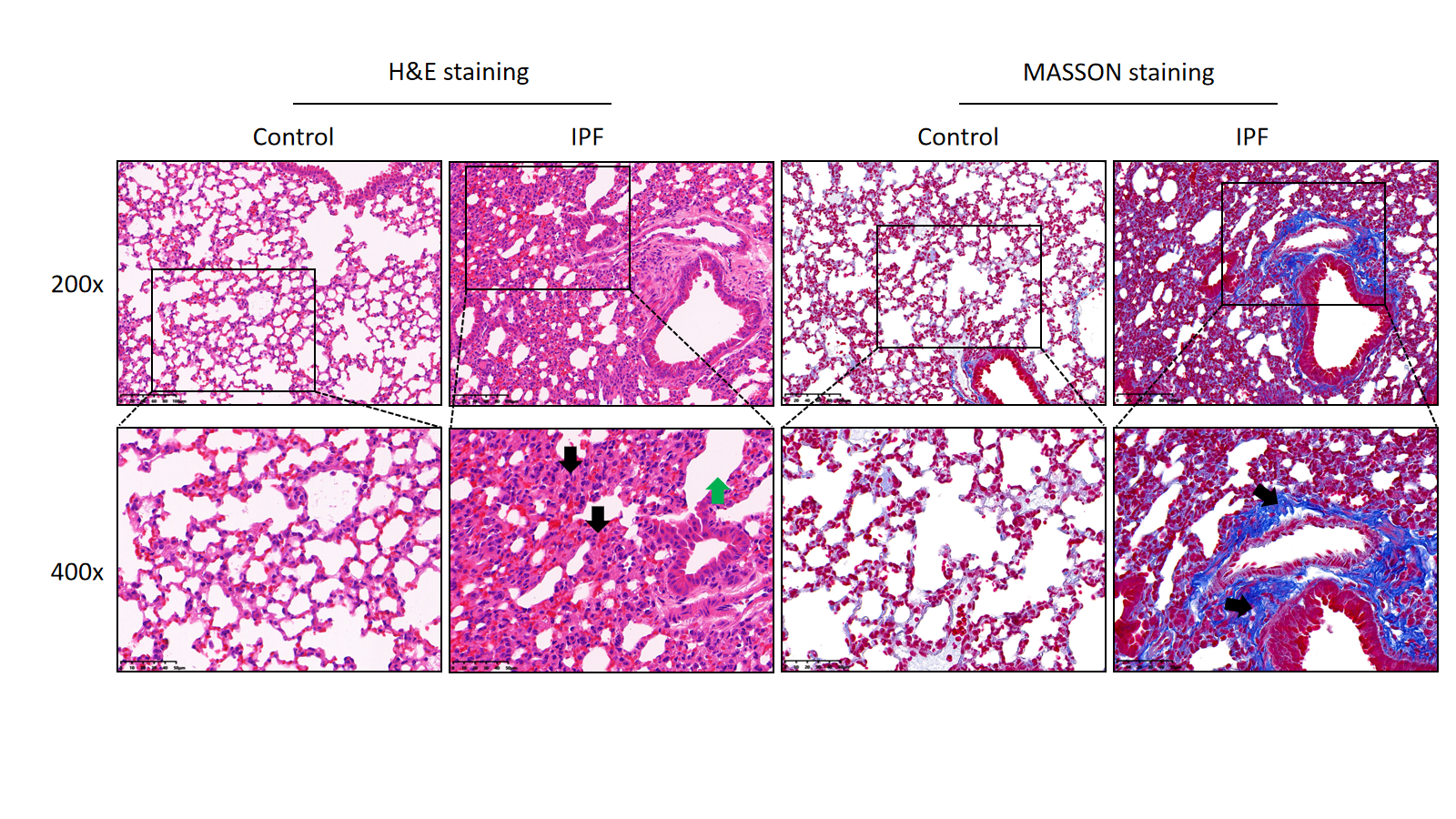

Supplement: Supplementary file 1 [file biology-15-00716-s001.zip › biology-4250175 Supplementary Files 4.28/Figure S1.tif]

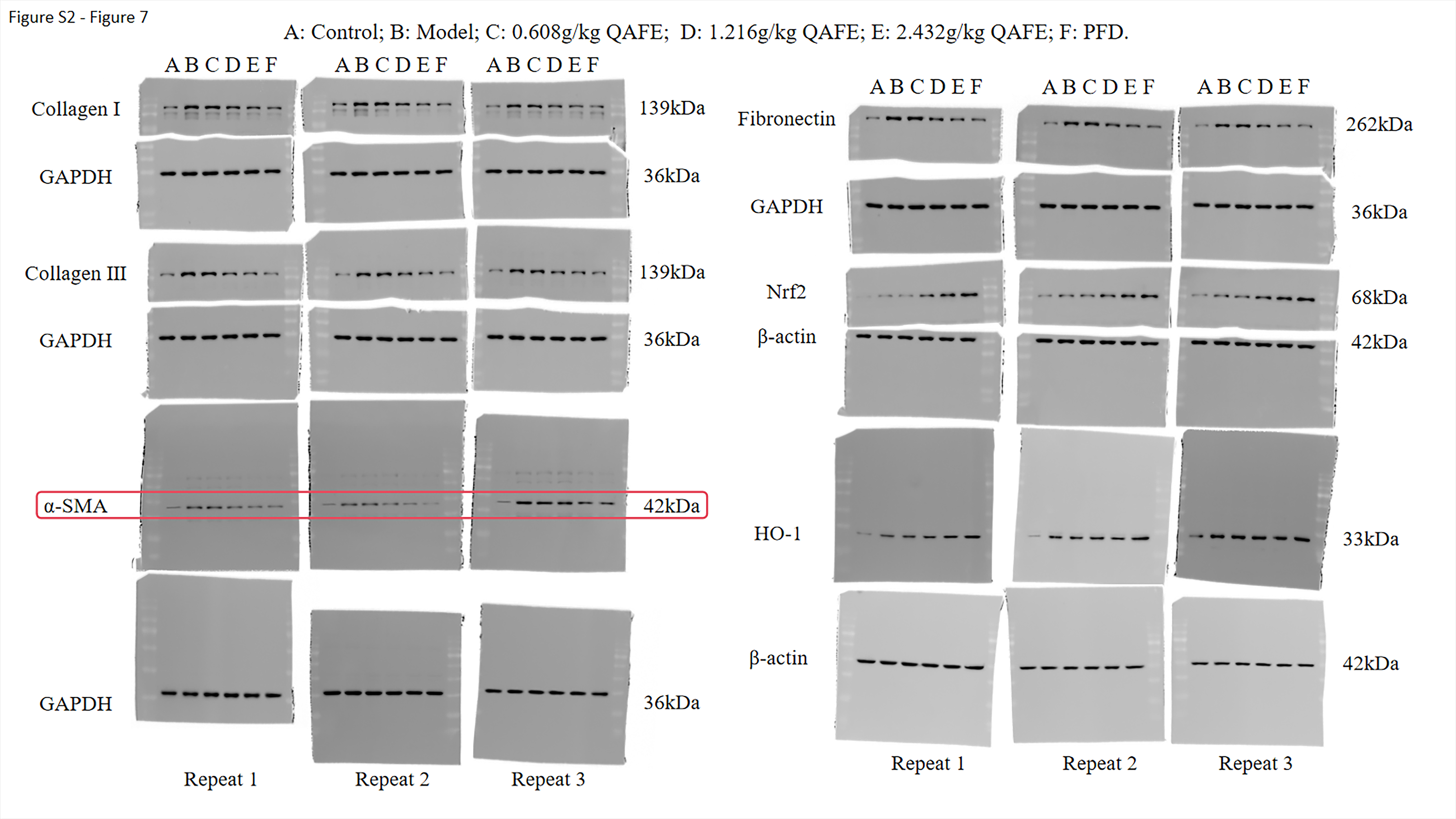

Supplement: Supplementary file 1 [file biology-15-00716-s001.zip › biology-4250175 Supplementary Files 4.28/Figure S2.tif]

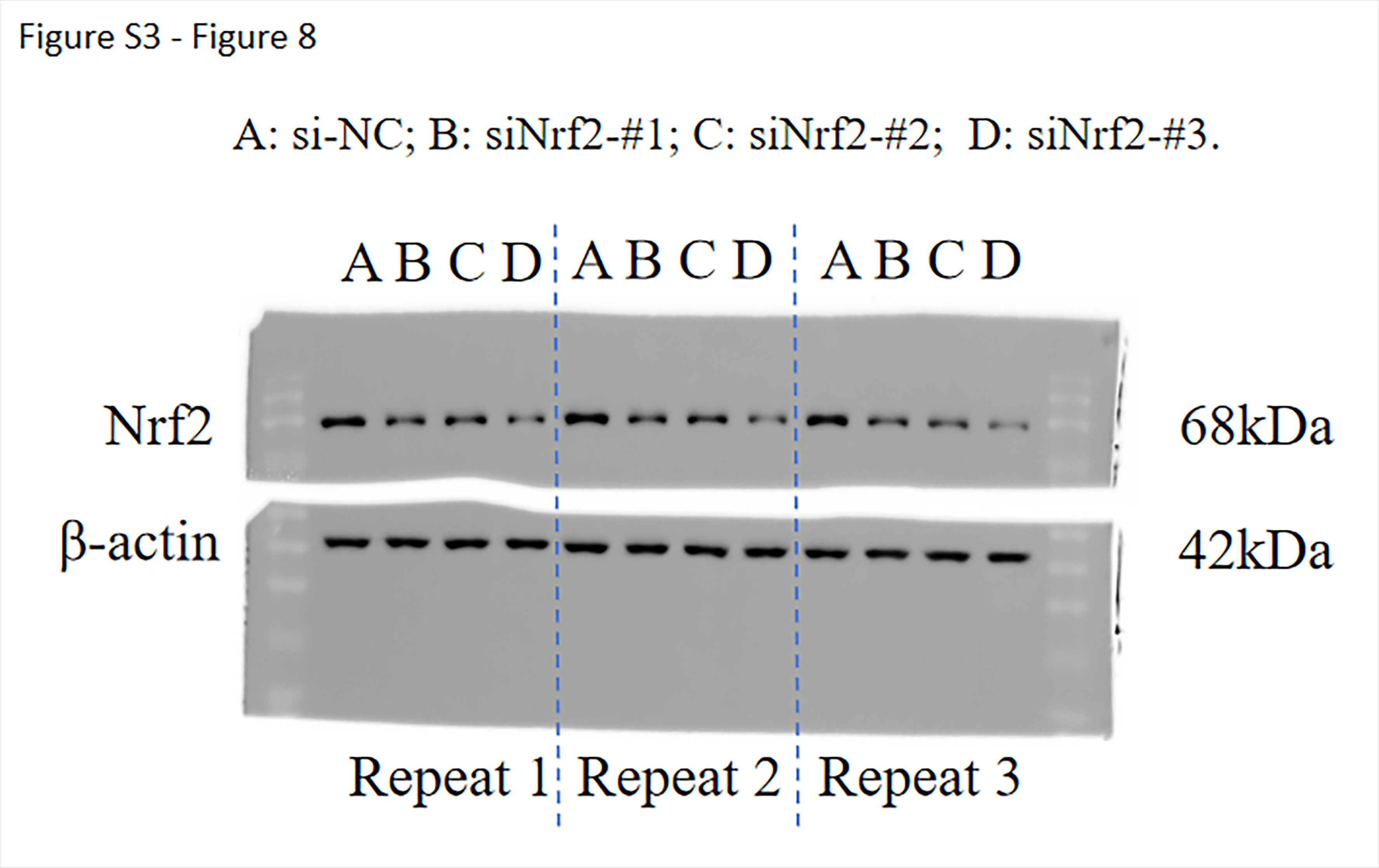

Supplement: Supplementary file 1 [file biology-15-00716-s001.zip › biology-4250175 Supplementary Files 4.28/Figure S3.tif]

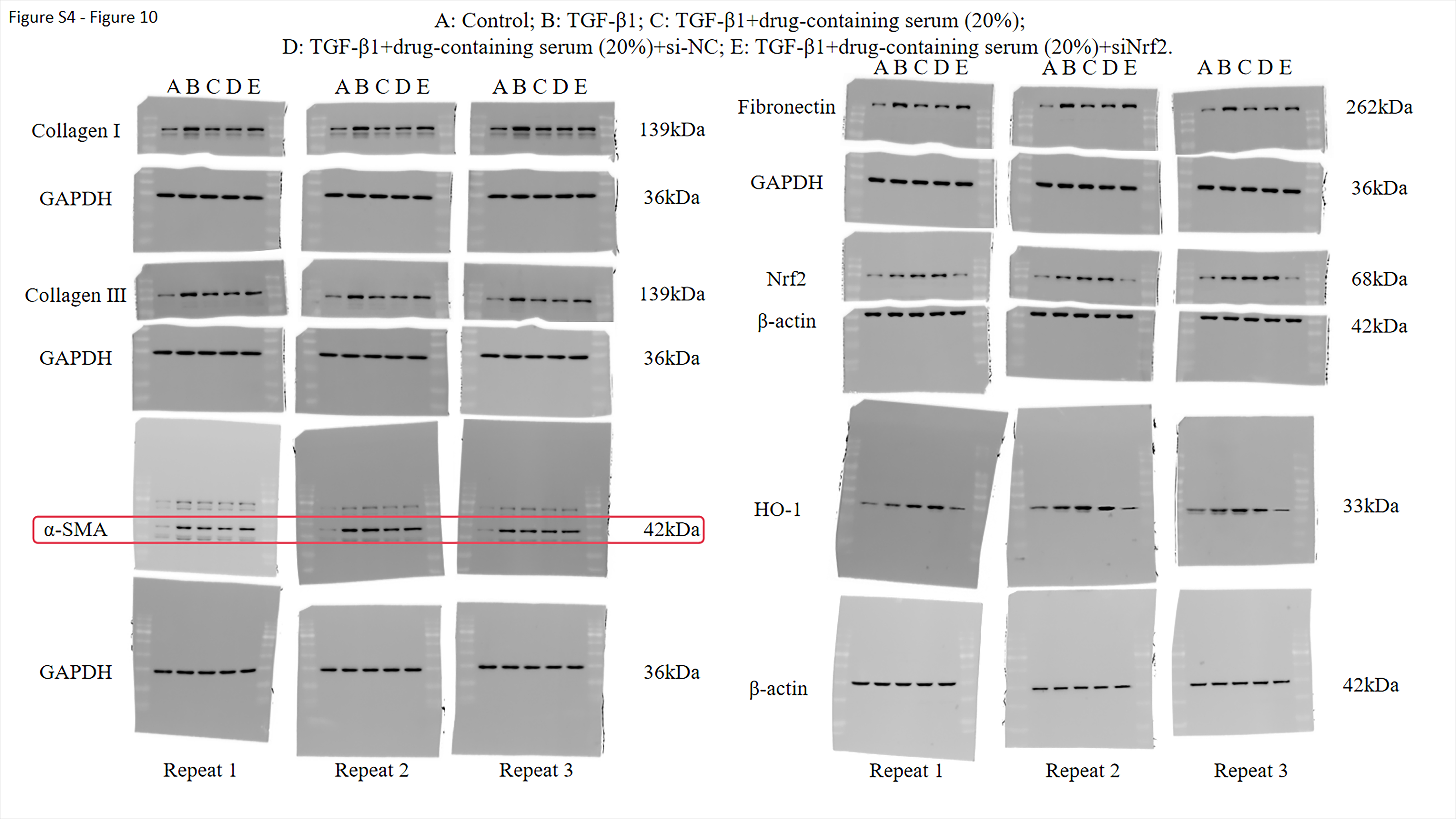

Supplement: Supplementary file 1 [file biology-15-00716-s001.zip › biology-4250175 Supplementary Files 4.28/Figure S4.tif]

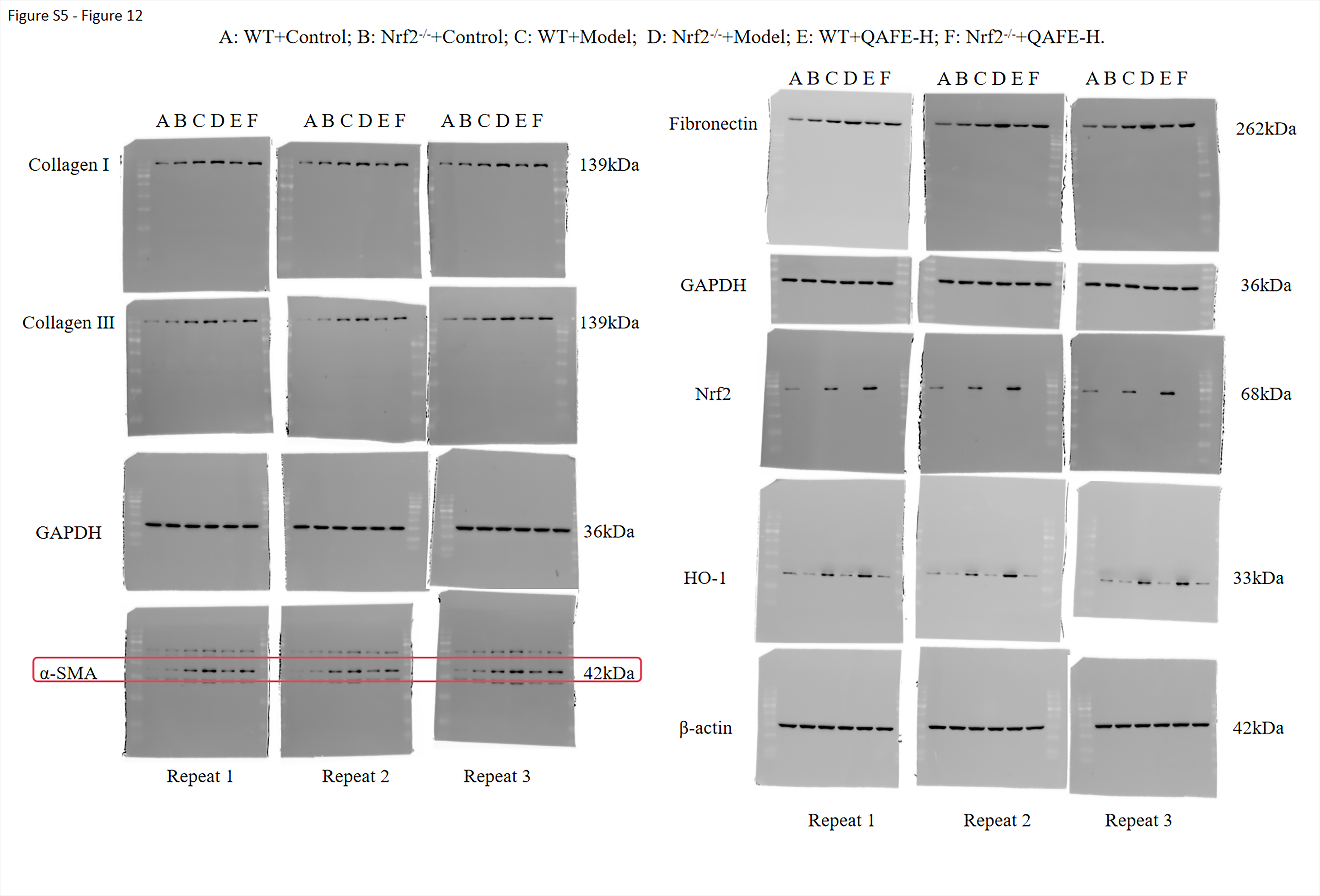

Supplement: Supplementary file 1 [file biology-15-00716-s001.zip › biology-4250175 Supplementary Files 4.28/Figure S5.tif]
